# Lithium inhibits NF-κB nuclear translocation and modulate inflammation profiles in Rift valley fever virus-infected Raw 264.7 macrophages

**DOI:** 10.1186/s12985-021-01579-z

**Published:** 2021-06-04

**Authors:** Raymond Tshepiso Makola, Joe Kgaladi, Garland Kgosi More, Petrus Jansen van Vuren, Janusz Tadeusz Paweska, Thabe Moses Matsebatlela

**Affiliations:** 1 Department of Biochemistry Microbiology and Biotechnology, School of Molecular and Life Science, University of Limpopo (Turfloop Campus), Sovenga, 0727 South Africa; 2 National Institution of Communicable Diseases (Special Viral Pathogen/Arbo virus Unit), 1 Modderfontein Rd, Sandringham, Johannesburg, 2192 South Africa; 3 CAES Laboratories, College of Agriculture and Environmental Sciences, University of South Africa, Private Bag X6, Florida, 1710 South Africa

**Keywords:** Macrophages, Inflammation, RVFV and NF-kB

## Abstract

**Introduction:**

Rift Valley fever virus (RVFV) is a zoonotic life-threatening viral infection endemic across sub-Saharan African countries and the Arabian Peninsula; however, there is a growing panic of its spread to non-endemic regions. This viral infection triggers a wide spectrum of symptoms that span from fibril illnesses to more severe symptoms such as haemorrhagic fever and encephalitis. These severe symptoms have been associated with dysregulated immune response propagated by the virulence factor, non-structural protein (NSs). Thus, this study investigates the effects of lithium on NF-κB translocation and RFVF-induced inflammation in Raw 264.7 macrophages.

**Methods:**

The supernatant from RVFV-infected Raw 264.7 cells, treated with lithium, was examined using an ELISA assay kit to measure levels of cytokines and chemokines. The H_2_DCF-DA and DAF-2 DA florigenic assays were used to determine the levels of ROS and RNS by measuring the cellular fluorescence intensity post RVFV-infection and lithium treatment. Western blot and immunocytochemistry assays were used to measure expression levels of the inflammatory proteins and cellular location of the NF-κB, respectively.

**Results:**

Lithium was shown to stimulate interferon-gamma (IFN-γ) production as early as 3 h pi. Production of the secondary pro-inflammatory cytokine and chemokine, interleukin-6 (IL-6) and regulated on activation, normal T cell expressed and secreted (RANTES), were elevated as early as 12 h pi. Treatment with lithium stimulated increase of production of tumor necrosis factor-alpha (TNF-α) and Interleukin-10 (IL-10) in RVFV-infected and uninfected macrophages as early as 3 h pi. The RVFV-infected cells treated with lithium displayed lower ROS and RNS production as opposed to lithium-free RVFV-infected control cells. Western blot analyses demonstrated that lithium inhibited iNOS expression while stimulating expression of heme oxygenase (HO) and IκB in RVFV-infected Raw 264.7 macrophages. Results from immunocytochemistry and Western blot assays revealed that lithium inhibits NF-κB nuclear translocation in RVFV-infected cells compared to lithium-free RVFV-infected cells and 5 mg/mL LPS controls.

**Conclusion:**

This study demonstrates that lithium inhibits NF-kB nuclear translocation and modulate inflammation profiles in RVFV-infected Raw 264.7 macrophage cells.

## Introduction

The immune system is the vertebrate-dependent phylogenetic defence mechanism against infectious agents such as toxins and microbes. It is classified into innate immune system and humoral immune system [1, 28]. Macrophages play a central role in the innate immune system and inflammation. These cells are antigen presenter cells (APC) located in various locations as tissue residential cells and also as circulating cells in the plasma [20]. The innate immune system is not entirely non-specific since myeloid lineage cells such as dendritic and macrophages express the pathogen recognition receptors (PRR) that recognise microbial components termed pathogen-associated molecular patterns (PAMP) [26].

Toll-like receptors (TLRs), Nucleotide-binding oligomerization domain-containing protein 1 (NOD1), NOD-2 and retinoic acid–inducible gene I (RIG-I)–like helicase receptors (RLRs) are PRR known to recognise microbial components and activate onset of various signalling pathways [1, 25]. Viral nucleic acids are recognised by a several receptors that include TLR-7 and 8 recognising viral single-stranded RNA (ssRNA), while RIG-I and TLR-3 recognise viral double stranded RNA (dsRNA). These molecules are expressed intracellularly on the endosome membrane. Viral glycol proteins are recognised by TLR 2 and 4, targeting mostly NF-κB linked inflammatory pathway and not the interferon regulatory transcription factor (IRF) 3/7 signalling pathway that produces anti-viral specific molecules such as type I IFN. The binding of viral ligands to the receptors recruit adaptor molecules such as myeloid differentiation primary response 88 (MyD88), Toll-interleukin 1 receptor domain-containing adapter protein (TIRAP) and TIR-domain-containing adapter-inducing interferon-β (TRIF) to the cytoplasmic domain of the receptors [1].

Toll-like receptor-3 (TLR3) induce the production of IFN via a MyD88-independent signalling pathway. TRIF is then recruited to dimerise with TLR-3 on the cytoplasmic domain. The pathway culminates with activation of IRF-3 and IRF-7 which translocate into the nucleus and bind to the IFN-stimulated response elements (ISREs), resulting in the expression of IFN-inducible genes. Conversely, TLR 7 and 9 induce the production of IFN via the MyD88 signalling pathway [1]. RIG-I is a cytoplasmic dsRNA detecting receptor which is not accessible to TLR-3. RIG-I interacts with IPS-1 through the CARD domain as its adaptor molecule. The resulting signalling cascade activates IRF-3 and IRF-7 in a similar manner used by TLR-3. The RIG-I detect the cytoplasmic replicating dsRNA while TLR-3 detect dsRNA in the apoptotic bodies of virally infected cells undergoing apoptosis [1].

Rift valley fever virus that causes Rift valley fever disease genome contains a negative sense single-stranded RNA genome made up of 3 segments namely the L segments that encode viral RNA dependent RNA polymerase, the M segments that encode envelop glycoproteins (Gn and Gc) a 78 kDa protein and a 14 kDa non-structural protein (NSm) [19]. Moreover, The S segments encode the nucleoprotein (N) in the negative-sense and a non-structural protein (NSs) in the genomic direction [19]. The non-structural protein NSs was shown to be the main virulence molecule and this protein has innate immune suppressive properties that aid in the viral replication and viremia [19]. RVFV NSs protein circumvents the innate immune system through inhibition of type I IFN (α and β) [19]. Other studies linked the NSs with RVFV pathogenesis through transcriptional shut down that lead to a weakened anti-viral response and IFNs production system [11, 27].

IFNs are important antiviral factors that stimulate antiviral molecules and recruit other immune cells to the inflamed site in order to limit viral spread. The type I IFNs have been shown to enhance the expression of protein kinase RNA-activation (PKR). In addition to the IFNs system, the PKR expression can be enhanced by dsRNA and ssRNA. The role of this serine threonine kinase is to phosphorylate eukaryotic translational inhibition factor 2 (eIF2), leading to the translational arrest of both cellular and viral mRNAs [7]. The PKR has shown some activity in the absence of NSs protein since NSs was shown to directly degrade PKR [7].

Monkeys that have shown high expression of IFNs cytokines have not developed RVF disease after exposure to RVFV. As a result, these cytokines were suggested to have the protective properties against RVFV. Animal model studies showed selective inhibition of the IFN-α since the production of IFN-γ, TNF-α, IL-6, IL-12, and IL-1 β was observed without detectable levels of IFN-α. Interestingly, IFN-γ and IL-12 have been suggested to lower viremia by stimulating natural killer cells (NK) cells and its cytotoxic role [19]. The evidence that NSs antagonise the IFNs cytokines makes RVFV infection difficult to clear. An in vitro study conducted by Jansen van Vuren and colleagues showed contradictory findings with those that postulate that NSs result in a transcriptional shut-down and weakened inflammatory response [11, 24].

The work by Jansen van Vuren et al. showed that immune response was mounted to a similar extent in both the fatal cases and non-fatal cases. This study showed that early infection samples have high levels of IL-8 and CCL-2/MCP-1 in serum as compared to sera from uninfected controls. Interestingly, fatal case serum has shown 10-fold increased levels of IL-6 (a pro-inflammatory cytokine) than non-fatal cases. The serum level of IL-10 in both early and late samples of the fatal and non-fatal cases were statistically not different. This study demonstrated that the fatality and survival of patients might rely on the extent of the regulation of inflammatory responses [24]. This immune deregulatory evidence reported by Jansen van Vuren et al. agrees with other publications, they shown severity that result with under-regulated or persistent inflammation [22].

The uncontrolled production of these inflammatory molecules has been linked to the pathogenicity of most of the chronic ailments such as neurodegeneration and cancer [8]. Wang and colleagues have linked the West Nile virus (WNV) infection consequences to elevated inflammation and damage to the endothelial integrity. This led to leukocyte extravasation, encephalitis and death [26]. It is, therefore the aim of the study to examine the regulatory properties of lithium on inflammation and NF-κB nuclear translocation in Raw 264.7 macrophages exposed to Rift valley fever virus.

## Materials and methods

### Cell culture and viral propagation

The RVFV AR 20368 strain was isolated in 1974 during the RVF outbreak in South Africa. The virus was propagated on Vero C1008 cells at an MOI of 0.2, followed by harvesting the monolayer after extensive cytopathic effect (CPE) was observed. The supernatant was stored at − 70°C after centrifugation at 3000 xg for 30 min [29]. The Raw 264.7 macrophage cells were obtained from Prof Lyndy McGraw (University of Pretoria, Photochemistry division, Onderstepoort campus). The cells were maintained in cell culture flasks at 37 °C, in a humidified 95% air and 5% CO_2_ atmosphere. Raw 264.7 cells were propagated in Dulbecco Modified Eagle Medium (DMEM) supplemented with 10% Fetal Bovine Serum (FBS), 2 mM L-glutamine and 1× penicillin-streptomycin. Vero C1008 cells were purchased from ATCC and propagated in Minimum Essential Medium (MEM), supplemented with 10% FBS, 2 mM L-glutamine and 1× penicillin-streptomycin (Lonza). Trypan blue dye and haemocytometer were used to determine cell density [14].

### Cell treatment

Lithium Chloride (LiCl) was purchased from Fluka (Chemika, Switzerland) and 500 mM stock was prepared and stored at 4 °C. Sodium Chloride was purchased from Sigma-Aldrich (USA) and the stock was prepared at 500 mM NaCl stored at 4 °C. The experiments were executed by seeding the Raw 264.7 cells at various densities depending on the experimental setting and then treated with these lithium concentrations (LiCl 2.5, 1.25 and 0.625 mM) as well as NaCl (2.5 mM).

### Determination of the cytokines expression pattern using ELISA

In an attempt to measure the production of the cytokines and chemokines, Raw 264.7 cell were seeded at 2.4 × 10^6^ cells for 3 h in T25 flasks. Thereafter  cells were inoculated with 10^4.8^ viral titre/mL for an hour. After one hour inoculation the supernatant was removed followed by treatment of cells with lithium concentrations (as outlined in cell treatment section) and LPS (5 mg/mL) for 24 h while the supernatant was collected in 3, 6, 12 and 24 h time intervals. The collected supernatant was frozen at − 20°C for later use. Enzyme-linked immunosorbent assay (ELISA) was executed according to the manufacturer’s protocol (Preprotech, USA). The capture antibody (for IL-10, IL-6, TNF-α, IFN-γ and RANTES) was diluted with PBS to 0.5 µg/mL and 100 µL was added to each well in a 96 well plate and incubated at room temperature (RT) overnight. The unbound excess capture antibody was aspirated and plates were washed with 300 µL wash buffer (0.05% tween-20 in 1× PBS) per well using ELx 405 auto plate washer (Bio-TEK instruments-Inc).

The standard was serially diluted 2 fold and 100µL was added in triplicates followed by addition of samples for 2 h. The added samples were aspirated and washed 4 times with wash buffer. The detection antibody was diluted in diluent to 0.5µg/mL and 100 µL was added in each well for 2 h at RT. This was then followed by aspiration of excess detection antibody and plates were washed 4 times with wash buffer. The 5.5µL avian peroxidase was then diluted 1: 2000 in 11 mL diluent, thereafter 100 µL of this diluted avian peroxidase was added to each well and incubated for 30 min at RT. This solution was aspirated and wells were washed 4 x with wash buffer. After blotting of wells, 100 µL of ABTS substrates solution was added to each well and the plates were incubated for colour development. Thereafter the colour was measured by reading the OD at 405 nm with ELx 802 universal microplate reader (Bio-TEK instruments-Inc)

### Determination of the production of reactive oxygen species (ROS)

The 2′,7′-Dichlorofluorescin diacetate (H_2_DCF-DA) is a cell-permeable reactive oxygen species (ROS) non-fluorescent probe. This molecule is deacetylated by cellular esterases and react with ROS to form a highly fluorescent 2′,7′-dichlorofluorescein. The Raw 264.7 cells were seeded at 4 × 10^5^ cells/well in a 6 well plate for 3 h and inoculation with RVFV at 1 × 10^4.8^ viral titre/mL for 1 h. This was followed by discarding excess virus and cell treatment for 24 h with lithium concentrations (as outlined in cell treatment) and LPS (5 mg/mL) followed. After 24 h of inoculation and lithium treatment, cells were stained with permeant H_2_DCF-DA at RT for 30 min in the dark and fixed with 3.7% paraformaldehyde for an hour. The fluorescent images were captured at 480 and 535 nm (Ex/Em) with EVOS FL Colour imaging system (Life technologies, USA). Moreover, the ROS production was measure quantitatively by seeding Raw 264.7 cells at 5 × 10^5^ cells/well in a 96 well plate for 3 h. This was followed by inoculation of cells with RVFV at 1 × 10^3.8^ viral titre/100 µL for 1 h. After 1 h of cell inoculation excess virus was removed and treatment of cells with lithium was executed as  in cell treatment section above for 12 and 24 h. After the 12 and 24 h incubation time H_2_DCF-DA was added for 30 min and then fluorescence intensity was measured at ex/em 480/535 nm using a Fluoroskan Ascent FL (Thermo Fisher Scientific, USA).

### Determination of the production of the reactive nitrogen species (RNS)

The 5,6-Diaminofluorescein diacetate (DAF-2 Da) is a cell permeant NO indicator that is deacetylated by intracellular esterase to DAF-2 that react with NO to yield a highly fluorescent triazolofluorescein (DAF-2T). The Raw 264.7 cells were seeded at 4 × 10^5^ cells/well in a 6 well plate for 3 h and inoculation with RVFV at 1 × 10^4.8^ viral titer/mL for 1 h. The excess viral inoculum was replaced with lithium treatment (as outlined in cell treatment) and LPS (5 mg/mL) for 24 h. After 24 h of inoculation, cells were stained with DAF-2 DA at RT for 30 min in the dark then cell were fixed with 3.7% paraformaldehyde for an hour. The pictures were captured with EVOS FL Colour imaging system (Life technologies, USA) at 480 and 535 nm (Ex/Em). For quantitative measure of NO, Raw 264.7 cells were seeded at 5 × 10^5^ cells/well in a 96 well plate for 3 h and then inoculated with RVFV at 1 × 10^3.8^ viral titre/100µL for 1 h. And then excess virus was removed and treatment of cells with lithium was executed as above for 12 and 24 h. After the incubation time, DAF-2 Da was added for 30 min and then fluorescence intensity was measured at ex/em 480/535 nm using a Fluoroskan Ascent FL (Thermo Fisher Scientific, USA).

### NF-κB translocation immunofluorescence assay

Raw 264.7 macrophage cells were cultured in 6 well plates on the slides at 4 × 10^5^ cells/well for 3 h. This was followed by RVFV inoculation at 1 × 10^4.8^ titre/mL for 1 h, and the excess virus was discarded. Cell treatment with lithium was executed as outlined in cell treatment and some wells were stimulated with LPS (5 mg/mL) for 24 h. The media was then aspirated after 24 h incubation and cells were fixated with 4% paraformaldehyde for 1 h. Thereafter, cells were permeabilised with 0.1% Triton X-100, 1%BSA for 60 min and the nonspecific binding sites were blocked by adding 1% BSA for 1 h, followed by 2× wash with wash buffer. Cells were incubated for 60 min with rabbit anti-p65 antibody (1:500) (Santa Cruiz, USA) followed by 3× wash with wash buffer. The cells were incubated with FITC-labelled goat anti-rabbit secondary anti-body for 60 min. After 5 min, the nuclear staining was done with DAPI, and then cells were mounted on slides using 50% glycerol and analysed using the fluorescent inverted Nikon Ti-E microscope at 20× magnification.

### Examination of inflammatory protein expression using Western blotting assay

In order to examine NF-κB related protein expression, the Raw 264.7 cells were seeded in T25 flasks for 3 h at a density of 1 × 10^6^ cell/mL and inoculation at 1 × 10^4.8^ titre/mL for 1 h, and the excess virus was discarded. Cell treatment with lithium was executed as outlined in cell treatment section and stimulated with LPS (5 mg/mL) for 24 h. After 24 h treatment cells were washed once with 1x PBS, thereafter, cell lysis was accomplished with 500µL lysis buffer (10 mM Tris-HCl, pH 6.8, 1% SDS, 100 mM sodium chloride, 1 mM EDTA, 1% NP 40, protease inhibitor), the cells were vortexed for 10 seconds and incubated on ice for 30 mins. The supernatant was collected by centrifugation at 15,000 ×g for 20 min at 4 °C and then the protein concentration was determined using BCA protein assay at 562 nm. For SDS PAGE 50µg proteins ware mixed with the sample buffer (1 mM Tris buffer pH 6.8, 20% SDS, 20% glycerol, 0.05% β-mercaptoethanol, 0.002% bromophenol blue) separated on 12% SDS-PAGE and then transferred to a polyvinylidene fluoride (PVDF) membrane using a semi-dry blotting system (Bio-Rad).

The membranes were blocked with Tris buffered saline (TBS) [150 mM NaCl, 50 mM Tris, 0.1% Tween, pH 7.5] containing 3% fat-free dried milk. The membranes were washed with wash buffer [0.05% TBS- Tween] and then incubated each time with 1:500 dilutions of anti- NF-κB-p65, IκB, HO-1, NOS-2 and β-actin primary antibodies for 1 h at RT. After incubation, the membranes were washed 3 x with wash buffer and incubated with corresponding peroxidase-conjugated secondary antibodies at 1:10 000 dilutions for 1 h at RT. The membranes were washed with wash buffer and the immune-reactive proteins were detected using the super-signal west pico chemiluminescent substrate (Thermo Scientific, Rockford, USA). The protein bands were visualised and photographed using the ChemiDoc XRS+ (Bio-RAD, USA).

#### Extraction of the cytoplasmic and nuclear proteins

In order to extract both the cytosolic and nuclear NF-kB protein, the Raw 264.7 cells were seeded in T25 flasks for 3 h at a density of 1 × 10^6^ cell/mL. The cells were treated and inoculated as outlined above. Cells were then washed once with 1x PBS and then harvested. For assessment of cytoplasmic proteins, buffer A composed of (10 mM HEPES, 10 mM KCl, 1 mM MgCl2, 5% glycerol, 0.5 mM EDTA, 0.1 mM EGTA, 1X protease inhibitor solution, 2 mM PMSF and 0.5 mM DTT) was added and the cell pallets were incubated on ice for 15 min with an addition of 0.5% NP-40. This was flowed by vortexing for 10 seconds and centrifugation at 12,000 xg for 1 min. For nuclear NF-κB protein extraction the centrifugation pellet was exposed to 500 µl high salt buffer B (20 mM HEPES, 1% NP-40, 400 mM NaCl, 10 mM KCl, 1 mM MgCl2, 20% glycerol, 0.5 mM EDTA, 0.1 mM EGTA, 1x protease inhibitor solution, 2 mM PMSF and 0.5 mM DTT) for 1 h on ice. The mixture was followed by vortexing for 15 sec and centrifuged at 12,000 xg for 1 min.

### Statistical analyses

All assays were performed 3 times in duplicates or triplicates and the error bars represent the degree of variance. Graph-Pad Prism 6 software was used to plot the graphs and statistical analysis was performed using GraphPad InStat 3 software. Duncan's multiple comparison t-test was used to determine significant differences between the means of treated and untreated groups. Differences were considered significant at *p ≤ 0.05; **p ≤ 0.01, ***p ≤ 0.001.

## Results

### Influence of lithium on the production of the inflammatory pro and anti-inflammatory Cytokines

Most viruses have developed mechanisms to evade the immune surveillance system which favours viral replication and increased viral progeny. RVFV is not an exception to this type of virus-induced immune invasion mechanism. RVFV is known to inhibit the innate immune system, particularly the type I IFN cytokine production as its mechanism of invasion [19]. Other studies [2, 24] outlined prolonged immune response as the primary detrimental factor in patients who suffer from this viral infection. This study examined the effects of lithium on cytokine and chemokines production after RVFV inoculation. This study has demonstrated that lithium stimulates INF-γ production in viral stimulated Raw 264.7 macrophage model system.

**Fig. 1:**
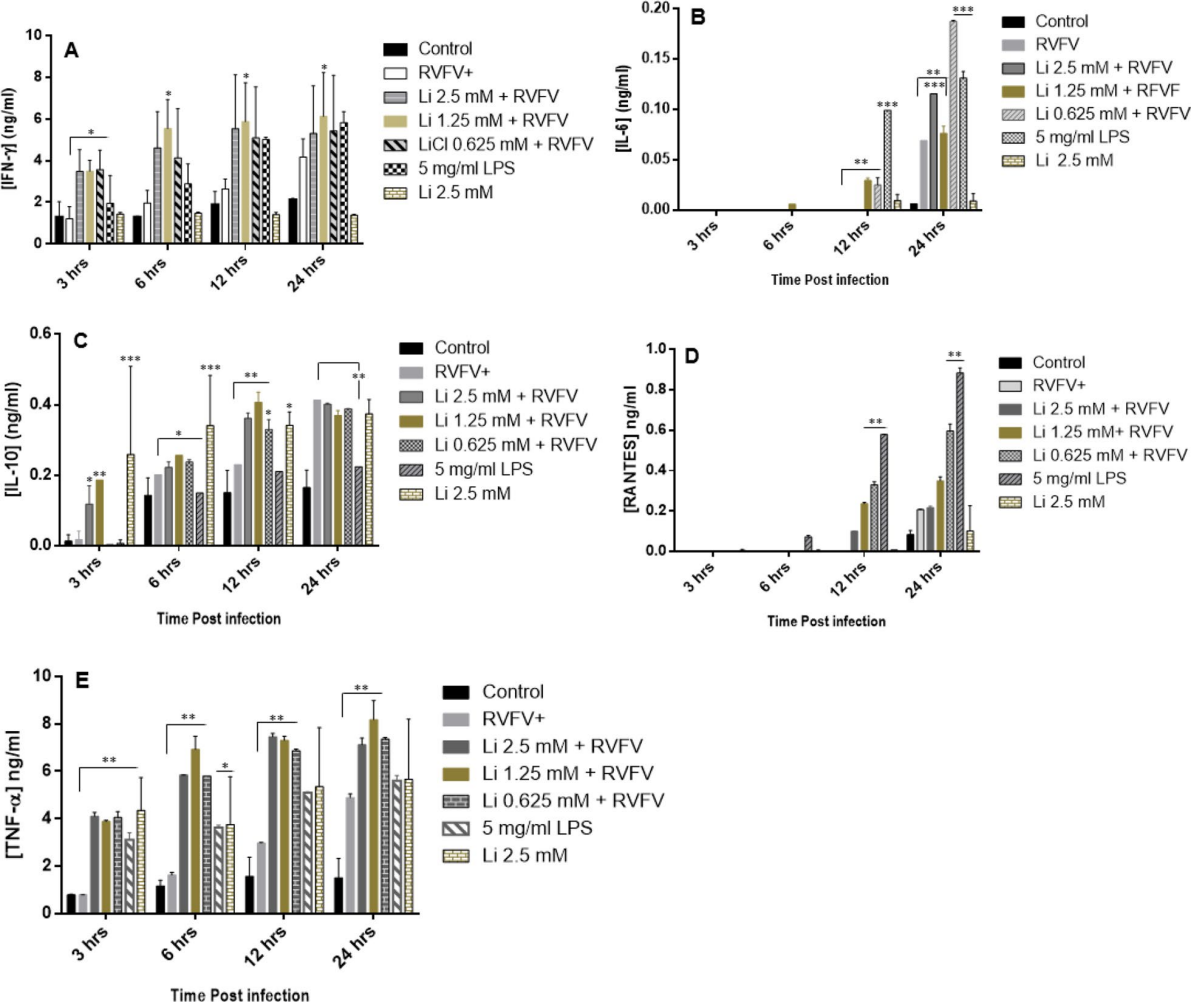
Determination of the effects lithium on TNF-α production after inoculation with the RVFV. In order to measure effects of lithium in TNF-α, IL-6, IL-10, IFN-γ and RANTES production, Raw 264.7 cells were seeded at the 2.4 × 10^6^ cells per T 25 flask for 3 h inoculated with 1 × 10^4.8^ viral titre/mL for an hour. The excess virus was washed and the cells were treated with various concentration of lithium and then the supernatant was collected in 3, 6, 12 and 24 h time intervals. The Sandwich Elisa (PeproTech, USA) was executed to measure the amount of these cytokines from various time points. The data points represent the mean + standard deviation (error bar). The plot was developed with Graph-Pad Prism-6 software and GraphPad InStat-3 was used to establish the statistical analysis

Lithium concentrations have shown to stimulate INF-γ production in RVFV-infected cells compared to the RVFV-infected control cells not exposed to lithium (Fig. 1a). The increase in INF-γ production also increased proportionally with an increase in incubation time, however, uninfected lithium treated cells did not stimulate INF-γ production. INF-γ production in RVFV-infected cells treated with lithium was even higher than LPS stimulated cells *albeit* reaching comparable levels at 24 h post inoculation (pi). Lithium at 1.25 mM induce the highest amount of INF-γ production compared to other concentrations in all the time points. Another prominent inflammatory cytokine, IL-6, was shown to be produced after 12 h pi (Fig. 1b). After 12 h (pi), 0.625 mM lithium produced IL-6 almost 3 fold compared to RVFV-infected cells compared to untreated RVFV-infected cells and 1.5 fold compared to LPS stimulated cells. The chemokine (RANTES/CCL-5) showed similar increment profiles to those observed in IL-6. Lithium induced CCL-5 production 12 h pi, the low concentrations of lithium stimulated more of CCL-5 (Fig. 1d).

Furthermore, lithium concentrations were shown to increase the production of the TNF-α compared to that of RVFV-infected control cells as from 6 h pi (Fig. 1e). The increase in TNF-α production was also shown to be time-dependent. The level of TNF-α after treatment with lithium was shown to be above that of 5 mg/mL LPS positive control. Lithium was shown to stimulate IL-10 (anti-inflammatory cytokine) production in both RVFV-infected and uninfected cells (Fig. 1c), from 3 h pi, the production of IL-10 increased with incubation time. The IL-10 was production more by 1.25 mM LiCl compared to other concentrations and control RVFV showed improvement as from 6 h pi and reached production peak after 24 h pi (Fig. 1c).

### Effects of lithium on the production of reactive oxygen and nitrogen species after RVFV inoculation

**Fig. 2:**
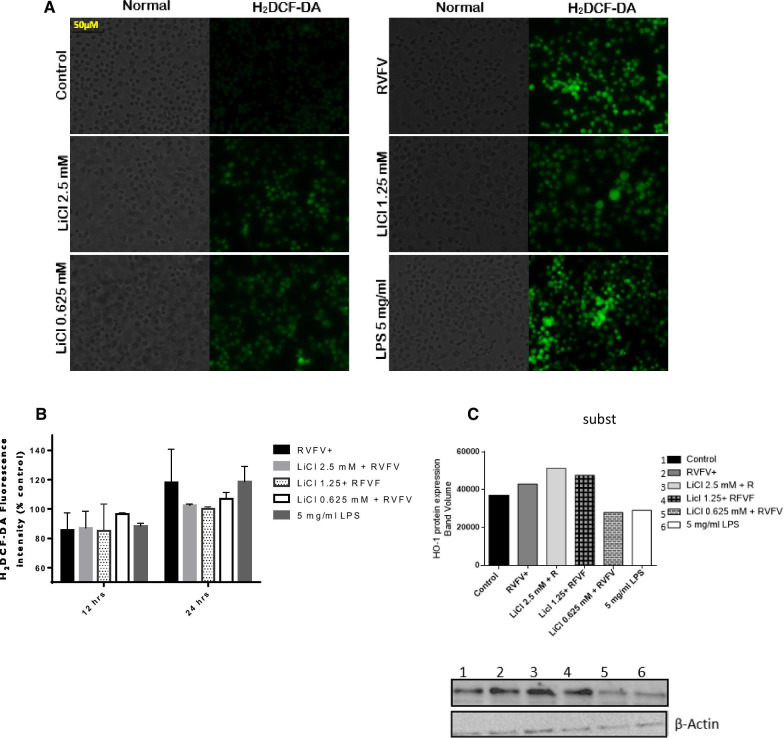
Determination of the effects of lithium on oxidative burst after Raw 264.7 cell are challenged with RVFV and expression of levels of the antioxidant enzyme. **a** Cells were seeded at 4 × 10^5^ cells/well in a 6 well plate for 3 h and then inoculated with 1 × 10^4.8^ viral titre/mL for an hour, the excess virus was substituted with fresh media and various lithium concentrations as well as 5 mg/mL LPS for 24 h. After 24 h of inoculation, cells were stained with H2DCF-DA at RT for 30 min in the dark and then cell fixed with 3.7% paraformaldehyde for an hour. The pictures where captured with EVOS FL Colour imaging system (Life Technologies, USA) Ex: 495 nm; Em: 515 nm. **b** Cells were seeded at 1 × 10^6^ cells/well in a 96 well plate for 3 h and then inoculated with RVFV at 10^3.8^ viral titter/100uL for an hour. The excess virus was then substituted with fresh media and lithium concentrations as well as 5 mg/mL LPS, this was incubated for 12 and 24 h. After the incubation hours the cells were stained with H2DCF-DA for 30 min in the dark, and then the fluorescence intensity was measured with Fluoroskan Ascent FL (Thermo Fisher Scientific, USA) at ex (485 nm)-em (538 nm). The graphs were developed with Graph Pad Prism-6 software and GraphPad InStat-3 was used to establish the statistical analysis. **c** In order to determine the expression of HO-1 protein, Raw 264.7 cell where seeded at 1 × 10^6^ cell/mL for 3 h and then inoculated with 10^4.8^ viral titre/mL for 1 h, and then the excess virus was substituted with a fresh media and lithium concentrations for 12 h. This was then followed by isolation of proteins and then western blotting assay. The pictures were captured with ChemiDoc XRS+ (Bio-RAD, USA)

**Fig. 3:**
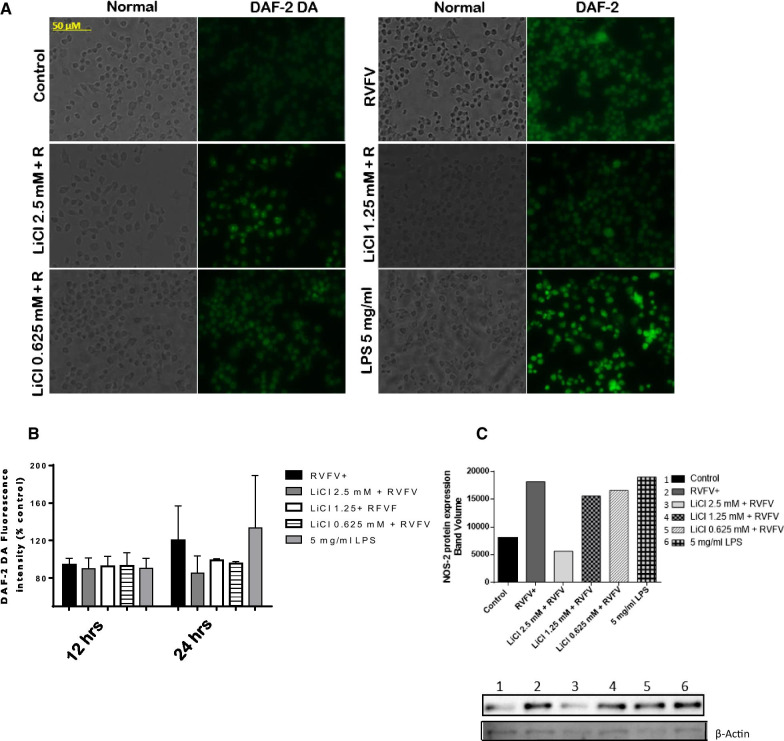
Determination of the effects of lithium on the production of inflammatory reactive nitrogen species 24 h post RVFV inoculation and expression levels of the NOS enzyme. **a** Cells were seeded at 4 × 10^5^ cells/well in a 6 well plate for 3 h and then inoculated with 1 × 10^4.8^ viral titre/mL for an hour, the excess virus was substituted with fresh media and various lithium concentrations as well as 5 mg/mL LPS for 24 h. After 24 h of inoculation, cells were staining with DAF-2 DA at RT for 30 min in the dark then cell were fixed with 3.7% paraformaldehyde for an hour. The pictures where captured with EVOS FL Colour imaging system (Life technologies, USA). **b** Cells were seeded at 1 × 10^6^ cells/well for 3 h and then inoculated with RVFV at 10^3.8^ viral titter/100 μL for an hour. The excess virus was then substituted with fresh media and lithium concentrations as well as 5 mg/mL LPS for 12 and 24 h. After the incubation hours the cells were stained with DAF-2 DA for 30 min in the dark, and then the fluorescence was measured with Fluoroskan Ascent FL (Thermo Fisher Scientific, USA) at ex (485 nm)-em (538 nm). The plot were developed with Graph Pad Prism-6 software and GraphPad InStat-3 was used to establish the statistical analysis. **c** In order to determine the expression of NOS-2 protein, Raw 264.7 cell were seeded at 1 × 10^6^ cell/mL for 3 h and then inoculated with 10^4.8^ viral titre/mL for 1 h, and then the excess virus was substituted with a fresh media and lithium concentrations for 12 h. This was then followed by isolation of proteins and then western blotting assay. The pictures were captured with ChemiDoc XRS+ (Bio-RAD, USA)

Reactive oxygen species (ROS) are generated as metabolic by-products during mitochondrial respiratory chain and excessively produced during inflammation [22]. In this study, the fluorescent probes such as 2ʹ,7ʹ–dichlorofluorescein diacetate (H_2_DCF-DA) and 4,5-diamino-fluorescein diacetate (DAF-2 DA) were used to determine the level of ROS and RNS production in virally infected cells. Figures 2a and 3a represent qualitative levels of ROS and RNS production in Raw 264.7 cells after treatment with lithium and infection with RVFV. Lithium concentrations are shown to downregulate the production of reactive molecules as the green fluorescence intensity is reduced in lithium treated cells. Quantitative measurement of fluorescence intensity (Figs. 2b, 3b) show that lithium lowered the production of ROS in a concentration-dependent manner. Highest inhibition of RNS production was observed in cells treated with lithium at 2.5 mM. These findings also showed that the production of ROS/RNS molecules only reached their highest levels at 24 hpi. Moreover, lithium concentrations are shown to upregulate antioxidant enzyme Heme oxygenase-1 (HO-1) (Fig. 2c). Lithium is shown to induce HO-1 expression in a concentration dependent manner.

The determination of RNS production using DAF-2 showed that lithium inhibited RNS production in RVFV-infected Raw 264.7 cells. Treatment of cells with lithium notably reduced DAF-2 fluorescence intensity. These observations were accompanied by elevation of nitric oxide synthase-2 (NOS-2) expression as depicted in Fig. 3c. The higher expression levels of the NOS-2 were elevated in control RVFV and LPS stimulated cells. Expression of NOS-2 was shown to be lowered by lithium in a concentration-dependent manner. NOS-2 is an enzyme that stimulates the production of the nitric oxide.

### Effects of lithium on nuclear translocation and expression of the NF-κB signalling pathway molecules

**Fig. 4:**
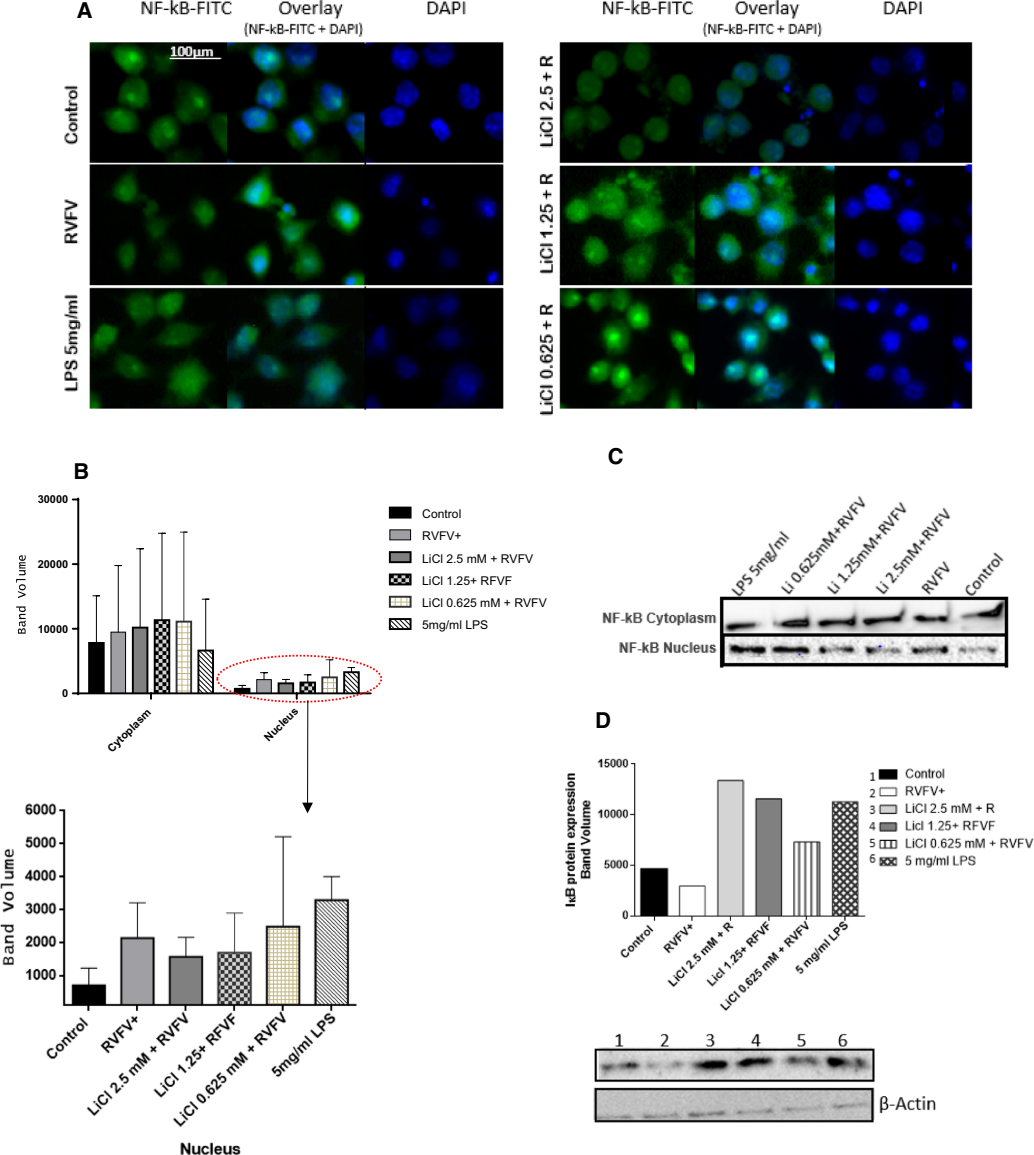
Effects of lithium on translocation of NF-kB between the cytoplasm and nucleus, and expression levels of IkB. **a** The cells were seeded at 4 × 10^5^ cells/well in a 6 well plate for 3 h and then inoculated with 1 × 10^4.8^ viral titre/mL for an hour, the excess virus was substituted with fresh media and various lithium concentrations as well as 5 mg/mL LPS for 24 h. After 24 h of inoculation, cells were fixed with 4% paraformaldehyde for an hour, and then permeabilised with (0.1% Triton X-100, 1%BSA) for 30 min. Permeabilised cells were incubated for 60 min with rabbit anti-p65 antibody (1:500). The primary antibody was followed by FITC-labelled goat anti-rabbit secondary Ab incubation for 60 min. Thereafter, nuclear staining with 25µg/mL DAPI was executed for 5min in the dark. Cells were mounted on slides using 50% glycerol and pictures were captured with fluorescent inverted Nikon Ti-E microscope at 20× magnification. **b **and **c** In order to determine the translocation quantity of the NF-κB, Raw 264.7 cell were seeded at 1 × 10^6^ cell/mL for 3 h in the T25 cell culture flasks and then inoculated with 1 × 10^4.8^ viral titre/mL for an hour, the excess virus was substituted with fresh media and various lithium concentrations as well as 5 mg/mL LPS for 24 h. This was then followed by isolation of cytoplasmic proteins as well the nucleus proteins and then western blotting assay followed. The pictures were captured with ChemiDoc XRS+ (Bio-RAD, USA). The ChemiDoc XRS+ image lab 5.2.1 software was used to measure band volume (Bio-RAD, USA). **c** The plot was developed with Graph pad prism-6 software and instat-3 was used to establish the statistical analysis. **d** In order to determine the expression of Ikb-α protein, Raw 264.7 cell were seeded at 1 × 10^6^ cell/mL for 3 h and then inoculated with 10^4.8^ viral titre/mL for 1 h, and then the excess virus was substituted with a fresh media and lithium concentrations for 12 h. This was then followed by isolation of proteins and then western blotting assay. The ChemiDoc XRS+ image lab 5.2.1 software was used to capture pictures and measure band volume (Bio-RAD, USA)

The molecular location of a transcription factor NF-κB is used as a molecular measure of inflammatory response. NF-κB binds to the kappa responsive element and induce expression of inflammatory mediators. Thus, the cellular location of the NF-κB molecule is an essential phenomenon in inflammatory studies. The molecular location of the NF-κB was examined using immunocytochemistry depicted in Fig. 4a. Figure 4a shows that NF-κB is located in both the nucleus and cytoplasm in lithium-treated cells, since green fluorescence is on both inside and outside of the nucleus. However, control untreated cells showed less of the green fluorescence in the nucleus and the image overlays showed an intense blue nuclear fluorescence, displaying that the two stains are not in the same location.

The positive control LPS (5 mg/mL) treatment shows the light blue nuclear fluorescence since all the green (NF-kB staining) and the blue (nucleus staining) are all in the same location in the nucleus. The control RVFV show similar patterns as control LPS, indicative of NF-kB nuclear translocation. On the other hand, Fig. 4b and c shows that lithium inhibits translocation of NF-κB with LiCl 2.5 mM showing the most inhibitory effects, although in this assay the inhibition margin is narrow. The IκB-α expression 12 hpi (Fig. 4d) showed to be elevated in lithium-treated cells in a dose-dependent manner. IκB-α is the inhibitory molecule that keeps NF-κB in the cytoplasm and is known to regulate the activity of this transcription factor [4].

## Discussion

The innate immune response plays a central role in the immune system as the first line of defence against foreign and infectious agents. Innate immunity, although non-specific, can orchestrate the humoral immune system through antigen presentation to the CD^+^4 T-cells [28]. It facilitates the first line of defence through inflammation, an ontogenetically old defence mechanism regulated by cytokines, products of the plasma enzyme systems, lipid mediators released from different cells, and vasoactive mediators released from mast cells, basophils, platelets and macrophages [23]. Macrophages are antigen presenter cells (APC) that produce cytokines as inflammatory mediators, which recruit other immune cells to the inflamed site and link the innate and adaptive immune response [15].

Despite the essential role played by inflammation, under-controlled inflammation leads to tissue damage and various adverse conditions that include neurodegeneration disorders, diabetes, cancer and endothelial leakage [8, 10, 26]. Viruses such as RVFV target macrophages to invade the innate immune system and use them as a vehicle to target tissues such as brain and liver [15]. This virus is known to inhibit the production of the IFNs as targeted by the NSs. This is thought to be the mechanism in which RVFV circumvent the immune system in favour of viral replication [19]. In addition to inhibited IFNs, NSs is shown to induce direct degradation and inhibition of the PKR involved in the translational arrest of both cellular and viral mRNA [7].

Weakened inflammatory responses by RVFV infection has been suggested to contribute to RVFV pathogenesis and fatality [19]. Contrary to this hypothesis, a recent body of evidence [2, 6, 24] suggests that deregulation and prolonged inflammation correlate with viral pathogenesis and fatality. The combination of this contradicting inflammatory evidence and the IFNs inhibitory role of the NSs led to the hypothesis that unbalanced and deregulated inflammation could be central to the RVFV pathogenesis and lethality. Our work examined lithium as a potential drug to restore regulatory patterns of inflammation and innate immune system. In this study, lithium with and without the viral stimulant has shown to stimulate the production of the primary pro-inflammatory cytokine, TNF-α, in Raw 264.7 cells as early as 3 h pi.

Analogous with these findings, [9] observed elevated TNF-α production in LPS-stimulated macrophages treated with lithium as early as 10 min post-stimulation, with the plateau reached within 12 h post-stimulation. Other studies hypothesised that lithium induced the production of TNF-α in macrophages subsequently this stimulate the production of the granulocyte-macrophage CSF (GM-CSF) from the endothelial cells. These observations can be associated with observed lithium-induced leukocytosis and granulocytosis as a result of GM-CSF [9, 16]. The secondary pro-inflammatory cytokine, IL-6, and a chemokine, RANTES, were shown to be produced during the late hours of infection 12 and 24 h with 1.25 mM LiCl being the most effective (Fig. 1b, d). These findings coincide with observations by [12] which showed that lithium did not induce significant production of the IL-6 in both stimulated and unstimulated cells.

Recent work on lithium-based inflammation studies reported inhibitory properties of lithium at 10 mM on RANTES production 24 h post stimulation with lipopolysaccharide (LPS) in a GSK-independent manner. This could suggest that the ability of lithium to modulate IL-6 and RANTES production is dose-dependent and that this cytokines production is NF-κB dependent, a pathway inhibited by lithium [13]. In vitro studies by van Vuren showed a 10 times elevated production of IL-6 in fatal cases as compared to non-fatal patients. This observation suggests that elevated production of this cytokine could be favouring virus survival as opposed to host defence [24]. Interestingly, lithium was shown to delay the elevated production of IL-6 pro-inflammatory cytokine in RVFV-infected cells. Another study showed that lithium enhances the production of another secondary pro-inflammatory cytokine IL-8 in both LPS and phytohemagglutinin (PHA) stimulated and unstimulated cells [12].

This current work demonstrated significant up-regulation of IFN-γ by lithium on RVFV stimulated cells as from 3 h post infection (Fig. 1a), however, lithium alone did not show any modulatory effects on this cytokine production. This work shows that lithium stimulates the production of some pro-inflammation cytokines which is the most important inflammatory phenomenon since Nfon et al. [19] linked the weakened inflammation with pathogenesis and lethality. A review by Nassar and Azab, supports the findings from this current study as they are in agreement with previous reports. However, a general view from a review by Nassar and Azab showed inhibitory role of lithium on various cytokines production rather than stimulation [18]. Nfon et al. [19] have linked elevated levels of IFN-γ to the survival of infected goats in animal experimental models. The IFN-γ is suggested to inhibit viral replication and stimulate the cytotoxic activity of the NK cells since lowered viremia has been observed in surviving infected goats [19].

Elevated IFN-γ levels could be another mechanism used by lithium to lower viral replication. In addition to pro-inflammatory cytokines, lithium stimulated the production of anti-inflammatory cytokine, IL-10 (Fig. 1c), in both viral stimulate and virus free lithium treated cells. Similar findings have been reported in other studies [12, 21]. These studies showed that lithium stimulates the expression of IL-10 and IL-1R anti-inflammatory molecules. This is suggested to be a regulatory mechanism as a result of the overwhelming production of inflammatory mediators known to have deleterious outcomes. Lithium has been shown to stimulate both the pro and anti-inflammatory cytokines in the current and previous studies [18, 12].

It is hypothesised that lithium could be restoring the balance in the production of inflammatory mediators, as pro-inflammatory molecules are later balanced by regulatory cytokines to limit over production of pro-inflammatory molecules. Besides the inflammatory properties of lithium observed in this study, lithium has been used for decades as a preferred treatment option for bipolar disorders despite the sparse and limited understanding of its mechanism of action [18]. Nontheless, under-regulated inflammation has been linked to pathological processes behind manic depression and bipolar disorders. Hence, studies suggest that lithium could be restoring inflammatory deregulation as the mechanism underlying its anti-depressant property [18, 12].

The RVFV-infected lithium treated cells have shown to lower production of the reactive oxygen and nitrogen species. The lowered production of these reactive molecules has been depicted in Figs. 2a and 3a, a qualitative assay. Quantitative findings (Figs. 2b, 3b) show the same trend as in the qualitative assay Figs. 2a and 3a. As represented in Figs. 1b and 2b, there is adequate production of these reactive species at 24 h pi. Previous work has shown lithium at 10 mM to reduce ROS production while 5 mM was effective in reducing NO production in LPS-stimulated Raw 264.7 cells [13]. More interestingly, in the current study, lithium downregulated the expression of NOS-2 enzyme (Fig. 3c), which correlate with low NO production. In addition to the inhibited NOS-2, lithium stimulates HO-1 expression, an antioxidant enzyme (Fig. 2c). This work aligns inflammation regulatory properties of lithium with activation of the NF-κB transcription factor.

Lithium-treated cells showed the presence of the NF-κB in both the cytoplasm and the nucleus, suggesting the reversal/inhibition of the transcription factor from translocating to the nucleus (Fig. 4a). Molecular translocation of NF-κB into the nucleus is observed to be lowered by lithium treated cells in a concentration dependent manner (Fig. 4b, c). Previous study [17] has shown that RVFV stimulate NF-κB nuclear translocation, culminating in production of inflammatory mediators and resulting in oxidative stress [17]. Oxidative stress is a condition emanating from excessive production of oxidants and free radicals, leading to imbalance between oxidants and antioxidants. Studies [3, 17, 22] have shown that oxidative stress conditions elicit biomolecules deformation that lead to altered cell function and then cell demise.

Narayanan et al. [17] hypothesised that the RVFV prevalent liver disease emanates from oxidative stress that leads to hepatic cell demise [17]. Inflammatory deregulation and oxidative stress have been linked with several pathogenic outcomes. This study suggests that lithium could ameliorate detrimental outcomes emanating from this viral infection. Figure 4d, show that lithium concentrations upregulate the inhibitory molecules, IκB-α. IkB-α inhibit the translocation of the NF-κB by masking its nuclear translocation domain [4]. Our previous work showed that high lithium concentration (10 mM) expressed the NF-κB inhibitors IκB-α, TRAF3, Tollip and NF-κB1/p50 to be lithium inhibition biomarkers [13]. What remains profound about lithium is that in as much as it was shown to promote expression of some pro-inflammatory cytokines, it stimulates anti-inflammatory cytokines in an attempt to avoid oxidative stress and nonspecific damage to host cell biomolecules.

**Fig. 5:**
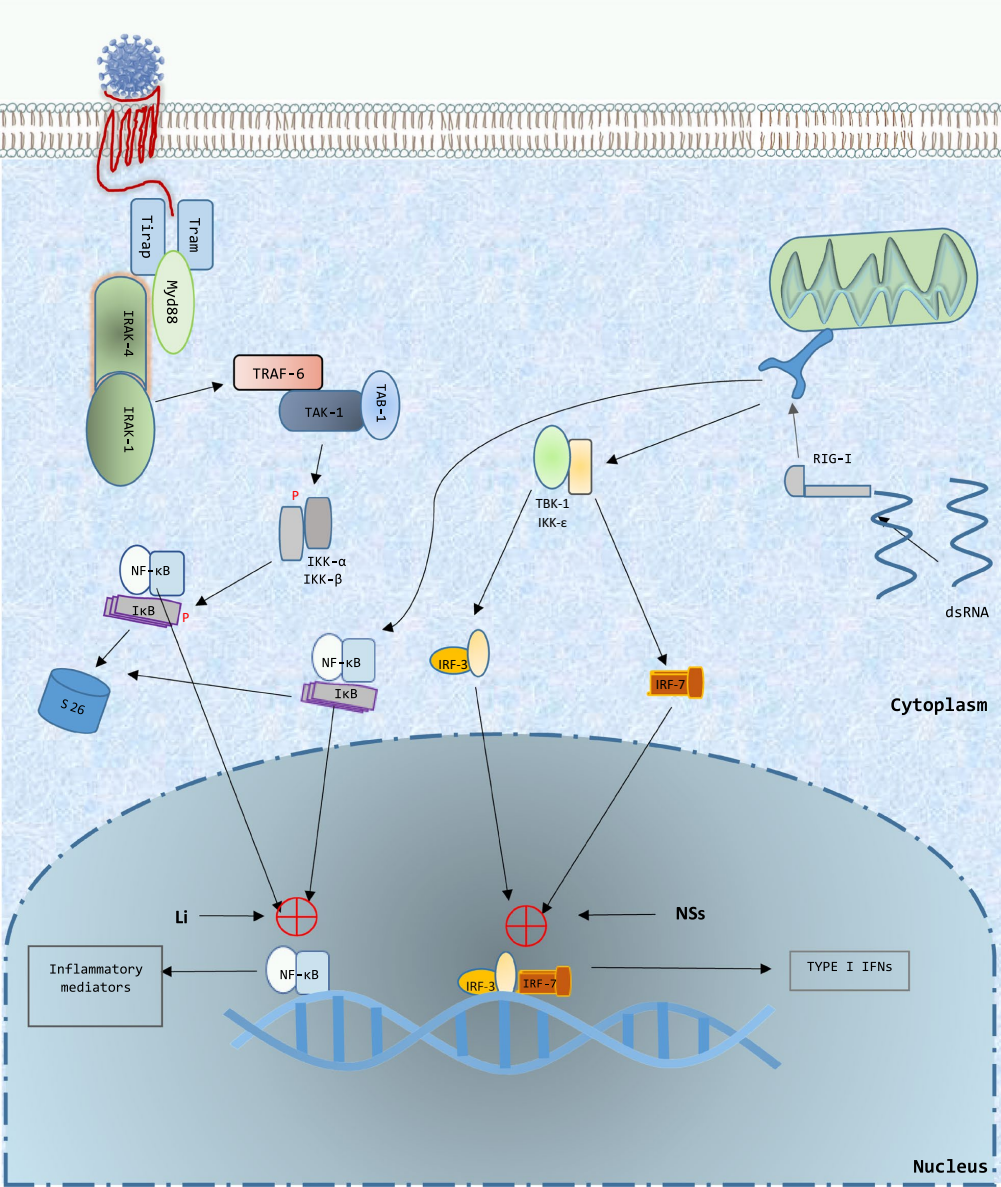
Determination of the canonical NF-kB and IRF3/7 signalling pathways and the effects of lithium post RVFV infection. TLR 2 and 4 are stimulated by the viral glycoproteins that in turn recruit adapter molecule Myd88 via tirap. The adapter molecules recruit Irak4 which phosphorylate recruit irak-1 which then associate with Traf-6. Traf-6 recruit Tak1 and Tab2. Tak1 phosphorylate IΚΚ-β, which then phosphorylate IkB which is then tagged for ubiquitination and then degradation by cytoplasmic proteases. This then allows translocation of NF-kB to the nucleus and inflammatory genes expression. The RIG-1 is known to be stimulated by dsRNA from replicating viral genome, which is said to be hidden from the TLR-3. This cytoplasmic receptor is shown to be essential for viral IFN expression. The RIG-1 is shown to associate with IPS-1 with its N-terminal card domain. The IPS-1 and RIG-1 association activate TBK1 and IΚΚ-ε which phosphorylate IRF-3 and 7. The NSs is suggested to interfere with the IFN signalling at the transcription factor level since there is an expression of other inflammatory mediators except for IFNs. Since, the NSs inhibit the interferon production via IRF inhibition other transcription factors such NF-kB continue to produce inflammatory mediators, hence, elevated production of other inflammatory mediators except the IFN. This diagram suggests that NF-kB inhibition as a result of upregulated IkB could be the, mechanism in which lithium restore dysregulated inflammation after RVFV infection leading to haemorrhagic fever pathogeneses observed during this viral infection

Previous studies showed that NSs selectively tempers with the type I IFN signalling while sparing the other signalling pathways that produces inflammation mediators such as ROS, NOS and Pro/anti-inflammation cytokines/chemokines. On a signalling level this could mean that NSs inhibit nuclear translocation of IRF3 and 7 transcription factors since they are central to type I IFNs production, or perhaps targeting the transcription of ISGs, leading to silencing of antiviral molecules as depicted in Fig. 5 [5]. Since NSs selectively inhibit IFNs which are linked to IRFs transcription factors, it then implies that other inflammatory mediators expressed by other transcription factors such as AP-1 and NF-κB will continually be produced leading to elevated inflammatory mediators and then oxidative stress. Thus, this work links the regulatory mechanism of lithium with inhibition of the NF-κB signalling pathway.

The NF-κB signalling pathway is suggested to be stimulated by the glycoproteins detected by TLR-4 or ssRNA detected by TLR-7 or dsRNA detected by the RIG-I. All these PRRs are linked to the NF-κB signalling pathway in as much as others stimulate IRF signalling as well (Fig. 5). The in vitro and ex vivo studies have shown cytokine and chemokines production excluding type I IFN during RVFV infection [19, 24]. Therefore, the activated NF-κB pathway continue producing these inflammatory mediators that are suggested to participate in the RVFV pathogenesis. Therefore, lithium restores the production of excessive inflammatory mediators as it has been observed to limit NF-κB translocation through upregulation of the IκB molecule.

## Conclusion

The NF-κB transcription factor is shown to be the targeted molecule behind the anti-inflammation properties displayed during RVFV infection. Results from this work show that lithium inhibits NF-κB-activity, which may be linked to the observed inhibited inflammatory mediators. Although additional work is required to outline the link between lithium and NSs. The current findings suggest that lithium could be used as an anti-haemorrhagic fever agent, since RVFV-induced oxidative stress, a suggested critical factor that lead to server symptoms such as haemorrhagic fever is shown to be reversed by lithium treatment. This study predicts that the RVFV virulence factor NSs inhibits the production of the type I IFN at a transcription factor level (IRF), since, the production of other inflammatory mediators such as RNS, ROS, cytokines and chemokines are observed except type I IFN. Lithium may potentially act as remedial agents for RVFV-induced by modulating NF-κB nuclear translocation in macrophages.

## Data Availability

The raw data are available upon reasonable request.

## Abbreviations

MyD88: myeloid differentiation factor 88
TRIF: TIR-domain-containing adaptor protein-inducing IFN-β
γ-GCS: γ- glutamylcysteine synthetase
FDA: US Food and Drug Administration
RNS: reactive nitrogen species
Tollip: toll interacting protein
TRAF3: TNF receptor-associated factor 3
HO: haem oxygenase
iNOS: inducible nitric oxide synthase
IκB-α: inhibitor of kappaB kinase alpha
MTT: 3-(4,5-dimethylthiazol-2-yl)-2,5 diphenyltetrazolium bromide
DAF-2DA: 5,6-diaminofluorescein diacetate
H_2_DCF-DA: 2',7'-dichlorodihydrofluorescein diacetate
GSK-3: glycogen synthase kinase-3
FBS: fetal bovine serum
TLR: toll like receptors
Tirap: TIR domain containing adaptor protein
Traf: TNF receptor-associated factor
NaCl: sodium chloride
RIG-1: retinoic acid-indicible gene 1
NSs: 78-kDa non-structural protein
LPS: lipopolysaccharide (gram-negative bacteria)
RTCA: real time cell analyser system (rtca)
TNF-α: tumor necrosis alpha
IFNs: Interferons
RANTES: regulated on activation, normal T cell expressed and secreted
IL-6: interleukine-6
IRF: interferon regulatory factor
NOS: reactive nitrogen species
ROS: reactive oxygen species
